# Evaluation of the diagnostic capacities of saliva sampling from pediatric patients: Protocol for a randomized intra-individual study

**DOI:** 10.1016/j.mex.2026.104038

**Published:** 2026-07-08

**Authors:** Laure Devienne, Laura Goram, Claire Morton-Fauche, Yohann Foucher, Antoine Pelras, Nicolas Lévêque, Aurélien Binet, Charlotte Bouchet, Christelle Pitard, Guillaume Beaumatin, Luc Deroche

**Affiliations:** a Paediatric medicine department, Centre Hospitalier Universitaire de Poitiers, Poitiers, France; b Unit for methodology and biostatistics, Centre Hospitalier Universitaire de Poitiers, Poitiers, France; c CIC 1402 Axe MDHP, CHU de Poitiers Service d’anesthésie-réanimation et médecine périopératoire, Université de Poitiers, Poitiers, F- 86000, France; d CHU de Poitiers, Laboratoire de Virologie et Mycobactériologie, Poitiers, F-86000, France; e Université de Poitiers, UR LITEC, Poitiers, F-86000, France; f Université de Poitiers, INSERM IRMETIST, Poitiers, F-86000, France

**Keywords:** Bronchiolitis, Respiratory distress, Saliva sample, Nasopharyngeal aspirate, Infant

## Abstract

Acute bronchiolitis is a common respiratory infection that affects approximately 30% of children under two years of age in France. While most cases are mild and treated on an outpatient basis, around 0.5% of children require hospitalization for monitoring and/or treatment. This condition, which is linked to various respiratory pathogens, accounts for a significant proportion of winter activity in hospital pediatric departments. Diagnosis for young children under two years of age is currently based on nasopharyngeal aspiration (NPA), performed by nurses on medical prescription. Although considered the gold standard, this technique is invasive and potentially traumatic for children. It often requires several caregivers and sometimes causes tension with families. The experience of the SARS-CoV-2 pandemic has highlighted the value of saliva sampling, with good diagnostic concordance with reference methods, as well as better tolerance and acceptability than nasopharyngeal swabbing in children over 2 years of age. In this context, our study aims to compare NPA and saliva sampling in the diagnosis of respiratory distress in children between 28 days and 2 years of age, to evaluate the concordance of results, and to compare tolerance and acceptability. Clinical trial number: NCT07350291.

## Graphical abstract

**Figure:**
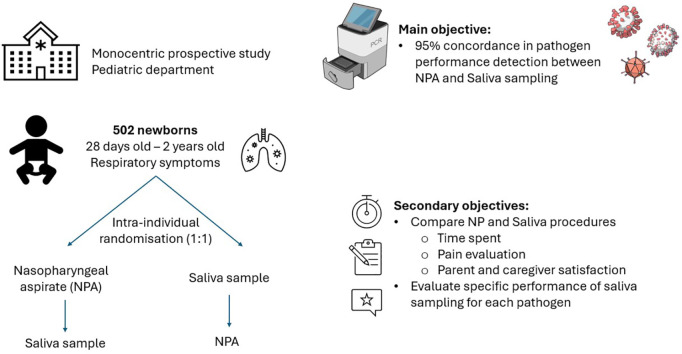

**Specifications table**

**Table:** 

**Subject area**	Medicine and Dentistry
**More specific subject area**	Pediatric respiratory virology
**Name of your protocol**	Evaluation of the diagnostic capabilities of saliva samples from pediatric patients with respiratory symptoms, promoting the active involvement of the individuals concerned and their families: a randomized, intra-individual study.
**Reagents/tools**	Saliva collection tube: ORACOL+ (Malvern Medical, Worcester, UK) Mucus suction device (40mL), suction catheter appropriate for the child's age (ch6/8) qPCR reagents: Alinity m Resp-4-Plex Assay (Abbott Molecular Diagnostics, Abbott Park, Illinois, USA); AllPlex Respiratory Panels Assays 2, 3 and PneumoBacter (Seegene Inc, Seoul, South Korea)
**Experimental design**	Bronchiolitis is a common viral respiratory infection in children under 2 years of age and is responsible for a significant number of hospitalizations. This study aims to evaluate the reliability and comfort of a noninvasive saliva sample for detecting respiratory pathogens in infants with bronchiolitis. The project will be based on intra-individual randomization (crossover) of 502 children at the University Hospital of Poitiers. Each patient will undergo both sampling techniques; randomization will determine the order in which the samples are taken.
**Trial registration**	This study was registered in the Clinical Trial Registry (NCT07350291)
**Ethics**	This research will respect the principles and foundations of the Helsinki Declaration and Good Clinical Practice. It will also be registered with a personal data protection committee in accordance with current French legislation prior to any recruitment. The results of this study will be disseminated through presentations at scientific conferences and publication in peer-reviewed journals.
**Value of the Protocol**	Most studies were retrospective. This proposal is the first to prospectively evaluate the feasibility and performance of saliva sampling for the etiological diagnosis of respiratory distress in children under the age of two. Randomizing the order of the sampling techniques will help reduce bias due to confounders, even if the first sample could influence the quality of the second. If diagnostic concordance with nasopharyngeal aspirate (NPA) is demonstrated, this method could offer a noninvasive and better-tolerated alternative, with several potential advantages: - For the child and their family: reduced pain, reduced parental stress. - For caregivers: reduced risk of splashing or contact with nasal secretions, greater acceptability of the procedure. - For healthcare organizations: speed, simplicity, and the possibility of use outside the hospital (community medicine, nurseries, population screening).

## Background

Bronchiolitis is a common viral respiratory infection in children under 2 years of age and is responsible for a significant number of hospitalizations each winter. While respiratory syncytial virus (RSV) is the most common agent, accounting for 50–80% of cases, other viruses have been recognized as responsible for bronchiolitis [1]. Indeed, since the rise of molecular biology, almost all common respiratory viral etiologies have been implicated in bronchiolitis, including human rhinovirus and enterovirus, human metapneumovirus, parainfluenza virus, seasonal coronaviruses and SARS-CoV-2, adenovirus, and influenza A/B, without clinical differentiation from RSV infection. Moderate cases require monitoring and oxygen or fluid support, while 2–6% of infants develop a severe form requiring intensive care. Unfortunately, no clinical score or clinical sign can predict the evolution of bronchiolitis. Diagnosis is based on clinical examination, usually accompanied by nasopharyngeal swabs (NP) or nasopharyngeal aspirate (NPA). Nasopharyngeal aspirate, generally performed in children under the age of two, is the most used sampling method but remains invasive, causes discomfort, and is time-consuming. This highlights the need for innovative approaches to improve the care and comfort of infants who must undergo virological testing. Salivary samples have been widely used during the SARS-CoV-2 pandemic in multiple settings, including infants and adults, healthcare workers, and symptomatic or asymptomatic patients [2,3].

In adults, saliva sampling is less invasive and can be self-collected, improving the acceptability of respiratory screening [3,4]. In children, saliva sampling was used during the pandemic for mass screening in schools and asymptomatic screening and can be either self-collected or retrieved with swabs or sponges [2,5]. While diagnostic performance was mainly evaluated for the detection of SARS-CoV-2, almost all other respiratory pathogens have been detected using saliva. These results were highly influenced by the type of saliva sampling (swabbing the oral cavity, drooling, using specific sponge-swabs for 30 seconds to one minute), the pre-analytical attention paid to saliva sampling (guidance on not eating, drinking, or performing oral hygiene 30 minutes before sampling; paying attention to a minimal volume collected), and the population (symptomatic or asymptomatic) [6,7]. Moreover, most studies were retrospective, and selection of patients was based on the results of NPA or NP swabs. Overall, the sensitivity and specificity of saliva sampling seem close, if not equivalent, to NP in adults. However, few studies have been conducted in children under the age of 2, specifically in the context of acute bronchiolitis.

Thus, the present study aims to evaluate the overall agreement of saliva sampling in children under 2 presenting to a university hospital with respiratory symptoms, compared to NPA. Secondary objectives are to evaluate the diagnostic performance of NPA versus saliva sampling for each respiratory pathogen, and to compare the reliability and comfort.

## Description of protocol

### Study design

**Fig. 1:**
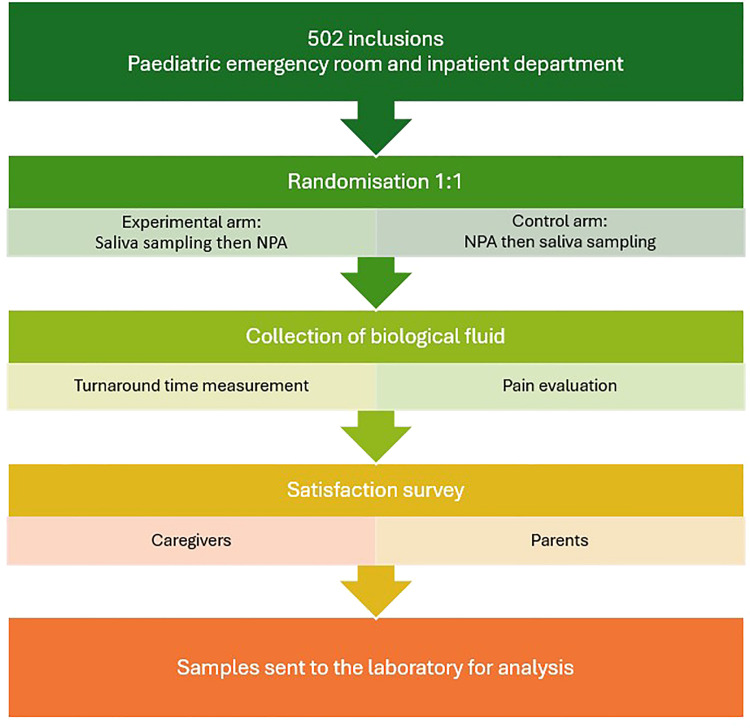
Study design. NPA: nasopharyngeal aspirate.

The PreSap study is a publicly funded, national, prospective, monocentric, randomized clinical trial comparing saliva samples and NPA in children between 28 days and 2 years old (Fig. 1). Once the protocol has been explained to the parents and child, and their agreement has been received, all patients will undergo the two sampling procedures after a randomization determining the order (crossover design with a 1:1 ratio). Randomization will be carried out with the REDCap software. Depending on the randomization, the saliva sample will be taken first or second, and the same will be done for the NPA. A rest period will be allowed between each sample so that the child can be calm again for the second sample. All temporal and behavioral data will be recorded.

### Study population

Recruitment is performed during a visit to the pediatric emergency department or during hospitalization in the pediatric department of the University Hospital of Poitiers, France. Inclusion criteria are: a) age between 28 days and 2 years; b) respiratory symptoms requiring an etiologic diagnosis as part of routine care; c) consent of both parents; d) affiliation with the social security system; e) no contraindication to either of the two sampling procedures (hemophilia, anticoagulant therapy, thrombocytopenia, risk of epistaxis, hypertrophic rhinitis, life-threatening emergencies, food, drink or oral care before the test (30 minutes), nasal antigen test already done in pediatric emergencies during this hospital stay and patient already included in the PreSap study).

### Ethical approval and registration

All procedures will be explained to the parents, and an informed consent form will be completed and signed. This study is registered in the Clinical Trial Registry (NCT07350291). The study protocol will be submitted to an institutional ethics committee and must be approved before any participants are enrolled.

### Experimental intervention

NPA will be performed as described in the recommendations of the French Pediatric Society [8]. The component used at Poitiers University Hospital is the Poly Medicure mucus extractor (mucus suction device with suction catheter) without transport medium. A minimum volume of 1.5 mL is required. This procedure is performed by a trained caregiver.

### Control intervention

Salivary samples will be obtained using a sponge swab (ORACOL+, Malvern Medical, Worcester, UK). These medical devices do not contain transport medium. Saliva should be collected without having eaten or drunk, or performed a mouthwash in the previous 30 min. Sponge swabs must be kept in the oral cavity for 30 sec to 1 min to obtain between 200 and 500 µL of saliva. This procedure can be performed by a caregiver or by the parents after they have been given the necessary instructions.

### Sending sample to the laboratory

All matched samples (NPA and saliva) will be sent at room temperature to the virology laboratory. If the analyses are not performed immediately, the samples will be stored at +4°C. The sponge swabs will be centrifuged as recommended by the manufacturer to collect saliva in the dedicated microtube.

### Primary outcome measure

To assess substitutability, the primary outcome will be overall agreement, i.e., a patient with a saliva-based test that diagnoses the same pathogens as the NPA-based test. Samples will be analyzed using the Alinity m Resp-4-Plex Assay (Abbott Molecular Diagnostics, Abbott Park, Illinois, USA) and AllPlex Respiratory Panels Assays 2, 3, and PneumoBacter (Seegene Inc., Seoul, South Korea). These assays allow the detection of flu A and B; SARS-CoV-2; RSV (A and B without distinction); adenovirus; enterovirus; metapneumovirus; parainfluenza (1 to 4 with differentiation); bocavirus (1 to 4 without distinction); coronavirus 229E; coronavirus NL63; coronavirus OC43; rhinovirus; *Bordetella parapertussis; Bordetella pertussis; Chlamydophila pneumoniae*; and *Mycoplasma pneumoniae*. All these assays use an exogenous internal control to ensure proper extraction, amplification, and the absence of PCR inhibitors.

Both samples will be analyzed at the same time to compare the results on fresh samples. Quantitative PCR analysis will be fully automated on all assays. However, only the NPA result will be visible to clinicians to avoid interfering with the study. In case of invalid results, samples will be tested a second time, possibly diluted if PCR inhibitors are suspected. All dilutions of a sample will be noted in the final analysis to ensure an unbiased comparison. Assays used in this study have not been approved for the saliva matrix, but use the same extraction protocol that has been validated on SARS-CoV-2 assays [9,10]**.**

### Secondary outcome measures

Secondary evaluation criteria are defined as follows:

a) Performance of saliva sampling compared to NPA for each pathogen detection (qualitative; semi-quantitative using cycle threshold (Ct) values).
b) Pain related to each sampling technique using the EVENDOL (EValuation ENfant DOuLeur; Evaluation Child Pain), a recognized and validated standardized pain assessment scale for children under 7 years of age [11]. It will be assessed by the caregiver.
c) Caregiver and parental satisfaction related to each sampling technique using a 0–10 visual scale.
d) Time spent by caregivers for each procedure (without considering the time needed for preparation and explanation).
e) Complications associated with the sampling techniques: bleeding, irritation, and exposure of healthcare workers to biological agents.
f) Procedural discrepancies associated with the two sampling techniques: involvement of multiple healthcare workers, repetition of the procedure, and cessation of care by the legal representative.
g) Non-conclusive results according to the sampling technique: insufficient sample volume, refusal of sampling, and laboratory measurement errors.

### Sample size

A review of the performance of saliva samples among adults and children by Laxton *et al*. reported concordance between saliva sampling and the reference comparator between 93 and 100%, depending on the studies and pathogens considered [12]. Accordingly, a 95% concordance is expected in our study. In the clinical context of bronchiolitis diagnosis, a 5% discordance (false-positive and false-negative results) is considered acceptable to validate the use of saliva samples instead of NPA. For a precision of 2% for the 95% confidence interval of the overall agreement (total interval length of 4%), it is necessary to analyze 457 patients. We estimate that a maximum of 10% of the children included will not be available for one of the two tests (insufficient sample volume, refusal of a second sample, etc.). It is therefore necessary to include 502 children.

### Data acquisition

Data acquisition will be carried out by investigators or clinical research assistants using electronic case report software. The following data will be collected: age (in months), sex, medical history requiring consultation or hospitalization, time since the onset of respiratory symptoms, Silverman score, fever, use of anticoagulants, time required for each respiratory sample (NPA and saliva), immediate complications of each respiratory sample, failure to retrieve one sample, number of caregivers required for each respiratory sample, help from parents required for one of the respiratory samples, procedural discrepancies, pathogens detected, cycle threshold value for each pathogen, satisfaction of caregivers and parents using a 0–10 visual scale, and pain assessed during each procedure using the EVENDOL scale.

### Data analysis

The type I risk will be 5% for all analyses. The 95% confidence intervals (CI) will be estimated by non-parametric bootstrapping (1,000 resampling), especially because of the low prevalences of pathogens and the related non-gaussian distributions. No interim analyses are planned. No correction of the multiplicity of tests will be performed. The analysis of the secondary endpoints will therefore be considered exploratory.

Because we expect a low percentage of missing values and missingness is at random, complete case analyses will be performed, i.e., patients with a missing value on the NPA-based test or the saliva-based test will not be considered in the analyses. The baseline characteristics (listed in the previous subsection) of the excluded patients will be compared with those of the analyzed population. If we observe differences, i.e., a non-random process, the outcomes’ analyses will be based on multiple imputation to reduce the risk of bias due to missingness (50 imputed databases, and confidence intervals obtained from the 5,000 resulting bootstrap samples).

*Descriptive analysis.* A description of demographic and clinical characteristics will be provided using means and standard deviations for continuous characteristics, or percentages and effectives for categorical characteristics. This description will be performed for the overall sample and according to the overall agreement (primary outcome) of the saliva-based and the the NPA-based tests.

*Analysis of the primary outcome.* We will estimate proportion of overall agreement and the related 95% CI. The agreement will be the diagnostic the same pathogens in-between the NPA-based and the saliva-based tests. More precisely, consider the following table for a pathogen where . The overall agreement is defined as

**Table:** 

Effective (proportion)		Saliva-based test		Total
		Positive result	Negative result	
NPA-based test	Positive result	()	()	()
	Negative result	()	()	()
	Total	()	()	()

*Analysis of the secondary outcomes (a).* For each pathogen, the following estimations and related 95%CI will also be estimated with the use of NPA-based results as gold standards [13]. The positive and negative percent agreement are respectively equaled to and . The Cohen’s kappa coefficient is, where . Additionally, by considering the NPA-based as the reference, sensitivity and specificity are .and, respectively. In addition, positive and negative predictive values are and, respectively.

*Secondary outcomes (b-d).* The pain level, the parental satisfaction, and the time spent by caregivers will be compared according to the sampling technique (NPA versus Saliva) in terms of means by paired two-sample t-tests. The interaction with the order (saliva/NPA or NPA/saliva) will also be explored by mixed linear models.

*Secondary outcomes (e-g).* The causes of complications, procedural discrepancies, and non-conclusive results will be described in terms of frequencies and percentages. In addition, to compare the two techniques, the following three proportions will be estimated and compared by using McNemar test: i) the proportion of sampling with at least one event among the following complications: bleeding, irritation, and exposure of healthcare workers to biological agents; ii) the proportion of sampling which has necessitated multiple healthcare workers, repeated procedure, or cessation of care by the legal representative; iii) the proportion of interventions with non-conclusive results: insufficient sample volume, refusal of sampling, or laboratory measurement errors. The interaction with the order (saliva/NPA or NPA/saliva) will also be explored by mixed logistic models.

### Strengths of the protocol

The present single-center, open-label, intraindividual trial will be carried out as soon as regulatory approvals have been obtained and will take place over three years at Poitiers Hospital, starting in winter 2026.

The literature is mostly composed of retrospective studies with potential selection bias due to the inclusion of patients who tested positive for a pathogen with the reference method [5,14]. Moreover, the sample sizes were often limited, as were the pathogens sought. The assessment of patients' pain and comfort was often neglected. The main strengths of our protocol compared to this literature are its focus on infants under 2 years of age, a population for which high-quality sampling is difficult to achieve. We propose a prospective crossover design in a clinical setting that enables a reliable performance comparison between the two procedures, limiting biases. If the primary objective is fulfilled, saliva sampling may be considered the first-line sample for respiratory diagnostics in children under 2 years in our hospital.

### Limitations of the protocol

The primary outcome is the overall agreement between the two tests. The primary outcome will be affected by the microbial epidemiology of respiratory infections, which may vary each year. Even if some tendencies can be predicted in seasonality, the emergence of a new viral lineage or variant may occur [1,15]. The proportion of atypical bacteria, such as *Mycoplasma pneumoniae* and *Chlamydophila pneumoniae*, is also unpredictable, as they are known to cause epidemics every 5 to 7 years (16). This issue could be partially addressed by the 3-year study design, which will limit the impact of epidemiologic variations.

The required sample size was calculated according to this primary outcome, whose agreement can be inflated by many double-negative samples. The other results, i.e., discordant and double-positive samples, will be important to consider to better appraise the usefulness of saliva-based tests. Nevertheless, the related confidence intervals could be large.

The single-center study design may also affect the findings, as the standard of care and procedures may vary from those of other hospitals. This study should be considered a pilot study and, if the primary endpoint is achieved, will be followed by a randomized, multicenter national study for definitive validation of the use of saliva sampling in children with bronchiolitis or respiratory symptoms in pediatric departments.

Some patients may be excluded from the study if one of the two samples cannot be obtained or analyzed (e.g., if the volume is too low for analysis). A 10% loss of samples has already been considered when determining the sample size to address this issue. Moreover, the saliva sponge swab that will be used in this protocol has already been widely used in several studies and has proven successful in recovering enough saliva for further analyses.

Our study aims to evaluate the opportunity to replace NPA, the current reference approach in clinical practice, by saliva sampling. Importantly, NPA does not represent an absolute gold standard.

Randomization will determine the order in which the two procedures (saliva sampling and NPA) are performed. The interaction effect of the order will be tested in statistical analyses, particularly for criteria relating to the time taken to perform the procedure and the level of pain or satisfaction (secondary outcomes). For reasons of feasibility and ethics, we cannot plan a washout period long enough to prevent this type of interaction. In the event of a significant interaction, the results may be presented conditional on the first sampling.

## Declaration of competing interest

The authors declare the following financial interests/personal relationships which may be considered as potential competing interests:

Laure Devienne reports financial support was provided by French Minister of Health, Families, Autonomy, and Disabled people. If there are other authors, they declare that they have no known competing financial interests or personal relationships that could have appeared to influence the work reported in this paper.

## Data Availability

No data was used for the research described in the article.
